# Role of stromal activin A in human pancreatic cancer and metastasis in mice

**DOI:** 10.1038/s41598-021-87213-y

**Published:** 2021-04-12

**Authors:** Georgina Mancinelli, Carolina Torres, Nancy Krett, Jessica Bauer, Karla Castellanos, Ron McKinney, David Dawson, Grace Guzman, Rosa Hwang, Sam Grimaldo, Paul Grippo, Barbara Jung

**Affiliations:** 1 Division of Gastroenterology and Hepatology, Department of Medicine, University of Illinois at Chicago, Chicago, IL USA; 2 Department of Medicine, University of Washington College of Medicine, 1959 NE Pacific Street, RR-512, Box 356020, Seattle, WA 98195-6420 USA; 3 Ronald Reagan UCLA Medical Center, UCLA Medical Center, Santa Monica, CA USA; 4 Department of Pathology, University of Illinois at Chicago, Chicago, IL USA; 5 Division of Surgery, Department of Breast Surgical Oncology, The University of Texas MD Anderson Cancer Center, Houston, TX USA; 6 Department of Surgery, University of Illinois at Chicago, Chicago, IL USA

**Keywords:** Molecular biology, Cancer, Gastrointestinal cancer, Pancreatic cancer

## Abstract

Pancreatic ductal adenocarcinoma (PDAC) has extensive stromal involvement and remains one of the cancers with the highest mortality rates. Activin A has been implicated in colon cancer and its stroma but its role in the stroma of PDAC has not been elucidated. Activin A expression in cancer and stroma was assessed in human PDAC tissue microarrays (TMA). Activin A expression in human TMA is significantly higher in cancer samples, with expression in stroma correlated with shorter survival. Cultured pancreatic stellate cells (PSC) were found to secrete high levels of activin A resulting in PDAC cell migration that is abolished by anti-activin A neutralizing antibody. KPC mice treated with anti-activin A neutralizing antibody were evaluated for tumors, lesions and metastases quantified by immunohistochemistry. KPC mice with increased tumor burden express high plasma activin A. Treating KPC mice with an activin A neutralizing antibody does not reduce primary tumor size but decreases tumor metastases. From these data we conclude that PDAC patients with high activin A expression in stroma have a worse prognosis. PSCs secrete activin A, promoting increased PDAC migration. Inhibition of activin A in mice decreased metastases. Hence, stroma-rich PDAC patients might benefit from activin A inhibition.

## Introduction

The survival rate for pancreatic cancer patients remains dismal, with an estimated 9% 5-year survival^1^. There is both a lack of early diagnostic tools and poor response to established treatments^2,3^. Consequently, patients often present with advanced disease and metastases^4,5^. Interestingly, it is one of the tumors with the densest peritumoral stromal involvement^6^. Most PDAC patients present with somatic mutations in *KRAS, TP53, CDKN2A and SMAD4*, although approaches to target these at the molecular level have not proven successful^2,7^. This may be due in part to a poor understanding of how the tumoral stroma interacts with the epithelial PDAC cells to support tumor progression, metastasis and resistance to chemotherapeutics^6,8^. Targeting the stroma is a new attractive approach in PDAC, but a better understanding of the mechanisms of stromal–epithelial interactions in PDAC is required.

Tumor stroma develops as a desmoplastic reaction to the chronic tissue damage caused by the tumor cells^9^. The stroma creates a heterogeneous tumor microenvironment containing several components including immune cells, capillaries, basement membrane, extracellular matrix and activated fibroblasts^10^ with the dominant component being the activated fibroblasts^9^. In pancreas, the pancreatic stellate cells (PSCs) are myofibroblast-like cells which can be activated by tissue injury and identified by their expression of α-smooth muscle actin (α-SMA)^11^. The activated PSCs proliferate and promote tissue repair^11^ through enhanced extracellular matrix (ECM) deposition, secretion of cytokines and growth factors, and contractile properties^9^. In the tumor microenvironment, the contractile properties increase tumor stiffness, leading to pressure-induced activation of latent TGFβ promoting epithelial to mesenchymal transition (EMT)^12^, which imparts enhanced mobility on the mesenchymal-like cancer cells^13^. Using colorectal cancer models, we have previously reported that TGFβ induces secretion of activin A, a TGFβ family member, from colon stromal cells, which promotes activin A-dependent migration of colon cancer epithelial cells^14^. Furthermore, we have shown that increased tumor microenvironment stiffness promotes activin A secretion from colon stromal cells, which enhances migration of colon cancer epithelial cells^13^.

Effects of Activin A are context-dependent as well as cell type-specific^15^ and involved in stem cell pluripotency and differentiation during development^16^, wound healing^17^, sex hormone regulation^18^, glucose homeostasis^19^ and maturation and activation of the innate and adaptive immune systems among other functions^20^. Evidence indicates that activin A signaling is important in a number of cancers including colorectal^14,21,22^, mammary^23^, lung^24^, esophageal^25^ and pancreatic^26^. Together these studies indicate that activin A is important in pancreatic cancer, but do not address the mechanisms by which activin A is increased or how it promotes pancreatic cancer metastasis. Here, we investigated the tumor microenvironment location and stromal cell involvement of activin A expression in advanced stages of PDAC using a human PDAC tissue microarray, an established murine pancreatic cancer model (KPC)^27^ and human pancreatic cancer cell lines. We utilized a neutralizing antibody against activin A for systemic inhibition of ligand-receptor interaction in tissue culture models and in mice with tumor burden to determine the impact on cancer cell migration and metastasis.

## Results

### Expression of activin A in the stroma, but not in the epithelium, correlates with reduced survival in PDAC

A previous study found that activin A serum levels were higher in pancreatic cancer patients compared to healthy subjects^26^, but the levels of activin A in tumor tissue has not been described. Using the Oncomine database^28^, we performed an in silico query into the activin A mRNA transcript (termed INHBA) expression and found a significant increase (p < 0.0001, fold change) in transcript expression in PDAC tissue compared to their matching adjacent-normal counterparts from each of the three studies (Table 1 and Figure S1A).

**Table 1:** Activin A gene expression is upregulated in pancreatic ductal adenocarcinoma (PDAC) or pancreatic cancer (PC).

Study	Number of samples		Fold change in gene expression	
	Normal	PDAC/PC	Normal	PDAC/PC
Badea	39	39	2.55 ± 0.72	22.02 ± 2.10
Pei	16	36	10.2 ± 2.93	43.46 ± 3.76
Langdon	5	10	0.42 ± 0.21	8.34 ± 1.53
Total	60	81	4.41 ± 1.01	28.80 ± 2.28

**Figure 1:**
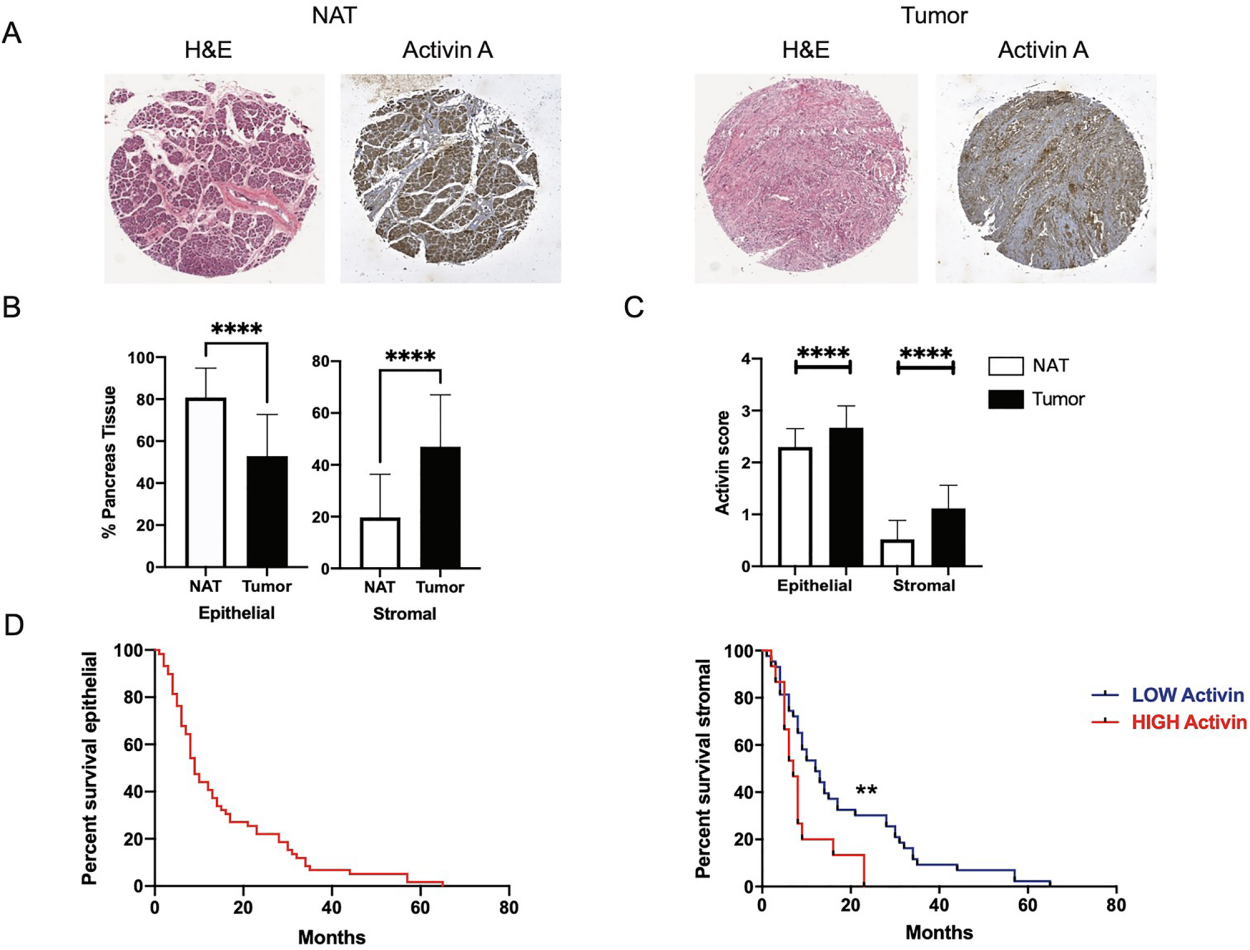
Activin A levels are significantly higher in tumor tissue and correlate to worse prognosis in PDAC. (**A**) Representative cores of pancreatic tumors from a human pancreatic tissue microarray stained with H&E to assess the percentage of epithelial versus stromal cells in each core. Adjacent sections were immunostained for activin A expression. (**B**) Quantification of the percentage of epithelial cells (left panel) or stromal cells (right panel) in normal tissue (white bar) versus tumor tissue (black bar). Statistical analysis is unpaired t-test. (**C**) Quantification of activin A in TMA (n = 63). An average of the percentage of epithelial and stromal fraction was calculated and scored in each TMA. Comparing epithelial (left panel) and stromal fraction (right panel) in normal tissue (white bar) versus tumor tissue (black bar). Statistical analysis 2-way ANOVA; mean ± SEM, ***(p < 0.001). (**D**) Kaplan–Meier plots of patient overall survival versus expression of activin A in either epithelial cells (left panel) or stromal cells (right panel). Statistical analysis Log-rank (Mantel-Cox) test, ** (p < 0.01), ***(p < 0.001).

To investigate if activin A protein levels are elevated in human PDAC tumor tissue, we performed histological immunostaining (IHC) on a tissue microarray (TMA) of human pancreatic cancer tissue and matching adjacent-normal tissue (n = 63, matching pairs) (Fig. 1A) where data on gender, age, stage, grade and survival were provided (Figure S1B). Normal pancreatic tissue is composed of acinar, ductal and islet cells, as well as connective tissue, primarily fibroblasts. However, as PDAC progresses, the tissue architecture becomes abnormal, there is increased metaplasia from acinar to duct-like cells and the surrounding tissue becomes more stroma-enriched with reduced parenchyma (Figure S1D). Quantification of the epithelial and stromal fractions (Fig. 1A) of all TMA cores showed a statistical difference in the ratio of epithelial/stromal fractions from adjacent normal to cancer cores; in normal cores, the epithelial fraction (73.24% ± 3.292, n = 54) was significantly higher (***p < 0.0001) than the stromal fraction (20.19% ± 2.461, n = 53), and the opposite was observed in cancer cores (epithelial: 2.321% ± 1.208, n = 56; stromal: 89.4%1 ± 1.885, n = 59) (***p < 0.0001) (Fig. 1B).

IHC staining for activin A protein expression levels was assessed in both epithelial and stromal compartments of tumor and adjacent-normal tissue by scoring staining intensity in a range from 0 to 3, where 0–1 indicates low activin A and 2–3 indicates high activin A (Fig. 1C and S1C). While there was no significant correlation between stage, grade, gender or age and activin A protein expression, activin A protein expression was significantly higher (***p < 0.0001) in tumor versus adjacent-normal tissue in both the epithelial and the stromal fractions of the tissue cores (Fig. 1C). There was a significant correlation with longer patient overall survival (OS) and low activin A stromal expression (p < 0.007). Patients with higher levels of stromal activin A staining had worse prognoses. In contrast, epithelial activin A expression levels were uniformly high and showed no correlation to disease outcome (Fig. 1D). These results highlight elevated expression of activin A in stromal cells of human pancreatic tumors in comparison to stromal activin A expression in normal tissue and the importance of its localization within the tumor microenvironment, rendering its inhibition in high activin A-expressing stroma-rich PDAC tumors an attractive therapeutic alternative.

### Activin A is secreted by pancreatic stellate cells, stimulating migration of epithelial cancer cells

**Figure 2:**
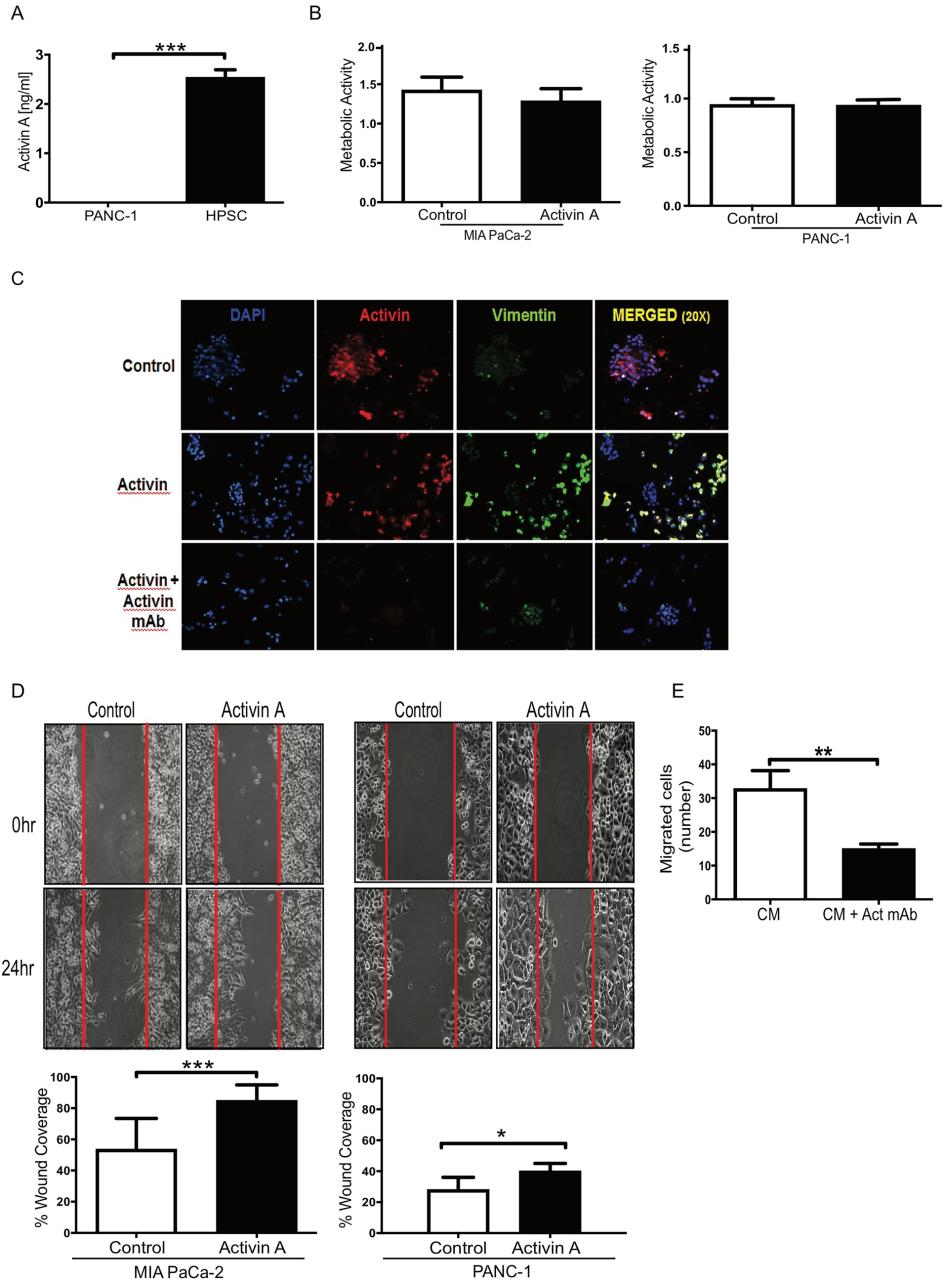
HPSCs secrete high quantities of activin A in vitro*,* significantly increasing migration but not metabolic activity of epithelial cancer cells. (**A**) HPSC and PANC-1 cells were plated at 75% confluency and serum starved for 48 h. Media was collected to determine secreted activin A levels. Data are graphed as mean ± SEM (n = 3). Comparison to standard curve showed HPSC secreted 2.537 ng/ml activin A, while PANC-1 secreted 0.009333 ng/ml. Statistical analysis unpaired Student t-test, *** (p < 0.001). (**B**) MIA PaCa-2 and PANC-1 cells were serum starved prior to treatment. Cell metabolic activity was determined as described in “Materials and methods” to indicate changes in cell number. Metabolic activity is expressed as mean ± SEM (n = 4 biologic replicates). Statistical analysis unpaired Student t-test. (**C**) PANC-1 cells were placed in chamber slides and treated with activin A (with/without anti-activin A antibody) for 24 h. Cells were fixed and incubated with indicated antibodies to detect activin A and vimentin by immunofluorescence. 10 × magnification. (**D**) MIA PaCa-2 (n = 3) and PANC-1 (n = 4) cells were serum starved prior to treatment. Activin A (25 ng/ml) or PBS (control) was added to the wells, and images were taken at 0 and 24 h (representative pictures). Experiment was performed in triplicate and percentages were averaged as mean ± SD. Statistical analysis unpaired Student t-test. *(p = 0.0005; p = 0.0438, respectively). (**E**) PANC-1 cells were placed in Boyden chamber with HPSC-conditioned media (with/without anti-activin A antibody) for 12 h after serum starvation. Migrated cells in the lower chamber was quantified by DAPI stained cells. Each experimental group was performed in triplicate and averaged (n = 4).

We previously reported that colon stromal cells secrete tenfold more activin A than colon cancer epithelial cells and, further, both activin A ligand and conditioned media from stromal cells stimulated epithelial cells to undergo epithelial to mesenchymal transition (EMT) and migrate^14^. Here we tested if Human Pancreatic Stellate Cells (HPSCs) similarly secrete activin A and activate PDAC cells to undergo EMT and migrate. Based on our observation of activin A expression in stromal cells of PDAC tissue cores (Fig. 1), we measured activin A levels in the media from an HPSC cell line and pancreatic PDAC cells (PANC-1) by ELISA assay. We found that HPSCs secreted significantly more activin A (2.537 ng/ml ± 0.1559, n = 3) than PANC-1 (0.009333 ng/ml ± 0.001202, n = 3), (270-fold increase) (Fig. 2A), suggesting HPSCs as a major source of activin A in the tumor microenvironment. To assess the effect of activin A on pancreatic cancer epithelial cells, we examined proliferation and migration in two different human cell lines, PANC-1 and MIA PaCa-2. Other established pancreatic cell lines were considered for these assays, however, our assessment required functional TGFβ signaling, which limited our pancreatic cancer cell line choice to those cell lines without mutations in the TGFβ signaling pathway. We measured metabolic activity as a surrogate of cell proliferation and observed that neither PANC-1 nor MIA PaCa-2 cells responded to activin A with a change in metabolic activity (Fig. 2B). Similar results were obtained using HPSC-conditioned media (Figure S2).

EMT is implicated in carcinogenesis and confers metastatic properties, including increased mobility, on cells^29^. We assessed the expression of an established EMT marker, vimentin, by immunofluorescence in activin A-treated pancreatic epithelial cancer cells, with and without anti-activin A antibody. This antibody prevents activin A interaction with the receptor by trapping the ligand; validation of the inhibitory effect of this approach was previously published^30^. PANC-1 cells treated with activin A alone had an increase in vimentin expression co-localized with activin A-expressing cells. Pre-treatment of PANC-1 cells with anti-activin A-neutralizing antibody blocked activin A staining and activin A-induced vimentin expression, suggesting vimentin expression increases in the presence of activin A (Fig. 2C). Future experiments will quantify these changes in vimentin expression.

To evaluate if activin A increases pancreatic epithelial cell mobility and migration, we assessed cell migration by wound closure assay and a Boyden chamber transwell assay. Both the PDAC cell lines, PANC-1 and MIA PaCa-2, responded to activin A with a significant increase in cell migration wound closure compared to control treatment, with a p-value of 0.0438 for PANC-1 and 0.0005 for MIA PaCa-2, respectively (Fig. 2D). We then incubated PANC-1 cells in transwell Boyden chambers with conditioned media from HPSCs, with and without an anti-activin A neutralizing antibody. Epithelial cancer cells with anti-activin A antibody migrated significantly less (15.00 cells ± 1.388, n = 30) than cells exposed to conditioned media alone (32.80 cells ± 5.366, n = 30) with p = 0.0022 (Fig. 2E). In sum, these data indicate that the pancreatic stromal cells secrete activin A, which in turn stimulates PDAC epithelial cells to undergo EMT with the increased mobility leading to cancer epithelial cell migration**.**

### Activin A plasma levels in murine PDAC models are elevated specifically in advanced disease

**Figure 3:**
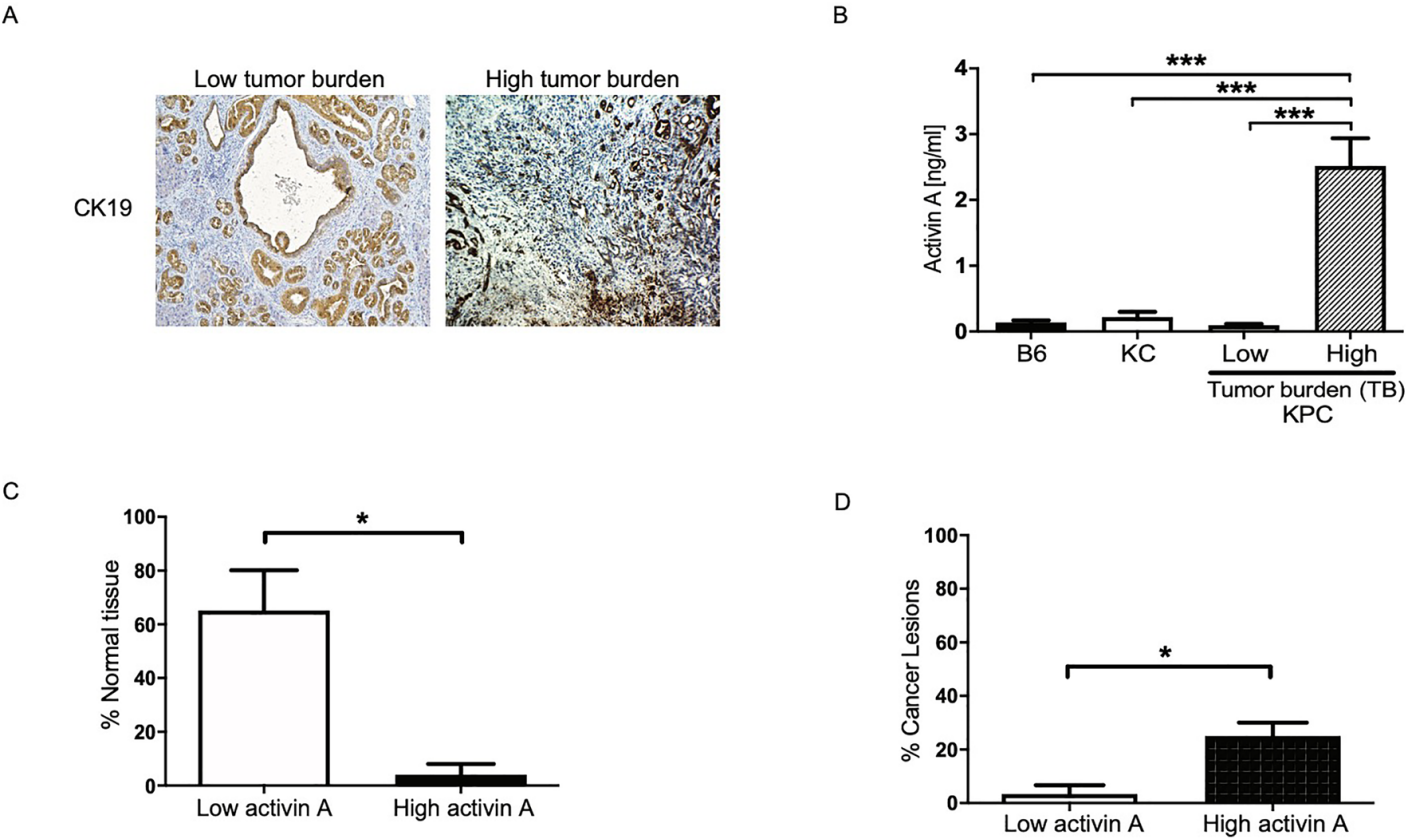
Serum activin A level is elevated in KPC mice with high tumor burden. (**A**) Immunohistochemical staining of cytokeratin-19 (CK19) in KPC mice indicates either focal staining in neoplastic lesions (low tumor burden) or diffuse extensive staining in cancerous tissue (high tumor burden). High tumor burden is defined as tumor weight equal or greater than 10% of body weight. (**B**) Serum activin A level in wild-type mice (B6 = C57BL6), neoplastic lesion model (KC) and PDAC model (KPC). *** (p < 0.001). Pictures taken at 10 × magnification field. Graph Bars expressed as mean ± SEM. Statistical analysis unpaired Student t-test. (**C**) Normal epithelial pancreatic tissue fraction in low (0 to 0.99 ng/ml) versus high (1.0 to 4.0 ng/ml) activin A expression in serum of KPC mice, *(p = 0.0158). (**D**) Cancerous pancreatic lesion percentage in low versus high activin A expression in serum of KPC mice, *(p = 0.0226).

Increased activin A in the serum of pancreatic cancer patients has been previously reported^26^ and increased activin A levels were found to correlate with disease severity. To understand if activin A levels in the plasma in murine models of PDAC mirror that of humans, we measured activin A secretion in plasma collected from PDAC murine models ranging in severity of disease from normal pancreas to neoplasia (KC mice) to cancer and metastasis (KPC mice). The KPC murine model of PDAC readily develops cancer with nearly 100% penetrance and can develop metastases, faithfully recapitulating human pancreatic cancer^31^. However, the level of tumor burden can vary widely across animals. Therefore, we first characterized the level of tumor burden in each murine model by staining murine pancreatic tumor sections for the expression of CK19, a marker of ductal cells, which is used as a marker for adenocarcinoma in the pancreas, with the level of staining indicative of tumor burden^32^ (Fig. 3A). The designation of low tumor burden was assigned when the animal had pancreatic intraepithelial neoplasms (PanINs) and the structures of pancreatic ducts and acini were maintained, as depicted in the left panel of Fig. 3A. High tumor burden was assigned when the tumor weight exceeded 10% of the body weight and tissues exhibited invasive disease, a loss of tissue architecture and a loss of epithelia with abundant stroma, as depicted in the right panel of Fig. 3A. Invasive disease was scored when ductal cells, which stain positive for CK19, were detected in the stromal compartment. These malignant cells, which display reduced polarity and odd nuclei, are found in small indistinct groups with no lumen and have a higher potential for migratory functions. Activin A secretion was quantified by ELISA from 6-month-old age-matched *wild type* mice that develop no disease (C57BL6 background), mice from models of pancreatic neoplasia (KC)^33^ and low versus high tumor-bearing KPC mice. We found significantly higher levels of activin A in the plasma of high tumor-burden KPC mice (2.514 ng/ml ± 0.4252, n = 4) compared to *wild type* (0.1363 ng/ml ± 0.03309, n = 12), KC (0.2170 ng/ml ± 0.08171, n = 6) and low burden KPC mice (0.0965 ng/ml ± 0.01900, n = 4) (Fig. 3B). The designations of low activin A expression (< 1.0 ng/ml) versus high activin A expression (1.0–4.0 ng/ml) were previously published by our group^34^. Here we assessed the lesions of KPC mice for percentages of normal epithelial versus stromal fractions and neoplastic versus cancer lesions in KPC pancreatic tissue relative to activin A expression levels (Fig. 3C,D). Mice with higher activin A expression had significantly smaller percentages of normal epithelium (H: 4.000 ± 4.000, n = 3 vs. L: 65.00 ± 15.00, n = 2) and more cancerous lesions (H: 25.00 ± 5.000, n = 3 vs. L: 3.333 ± 3.333 n = 3) than KPC animals with low activin A expression. These data suggest that activin A levels are specifically higher in advanced murine PDAC disease and correlate to tumor burden, loss of normal epithelia and increased cancerous lesions.

### Inhibition of activin A in an in vivo PDAC model leads to reduction in metastasis

**Figure 4:**
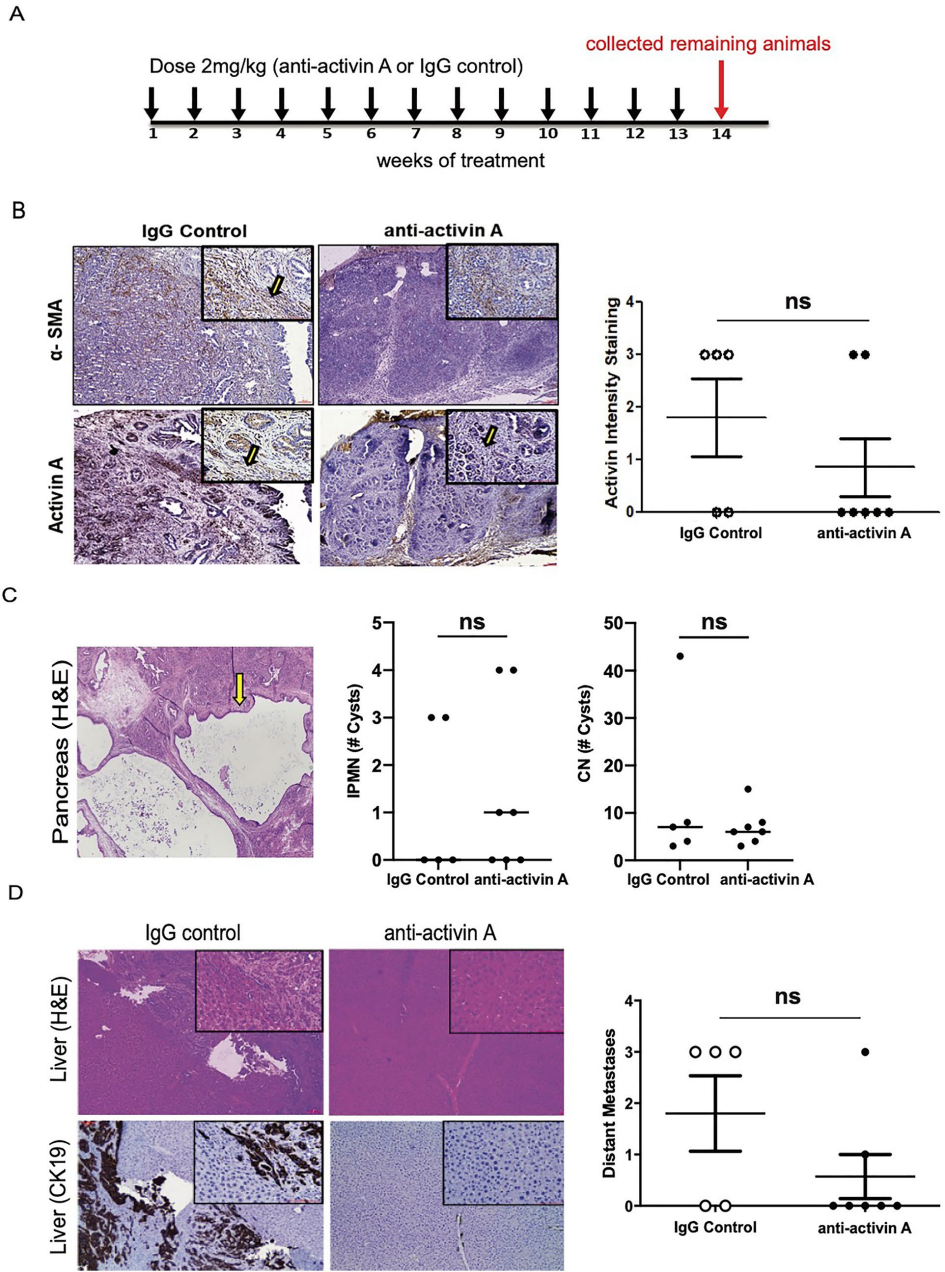
Inhibition of activin A in KPC mice significantly reduces metastasis. (**A**) Time course of anti-activin A neutralizing antibody injected in mice weekly (2 mg/kg). Control animals were injected with the same concentration of IgG isotype. Remaining animals were collected at the end of week 13 of treatment. (**B**) Representative image (×4 magnification) of H&E staining of pancreas from control treated (IgG) and anti-activin A treated KPC mice IHC-stained for either α-SMA (to indicate stromal cells) or activin A. Representative stromal cells marked by arrows. (**C**) Representative image (×4 magnification) of H&E staining of pancreas used for quantification of CN (yellow arrow) cystic lesions. Quantification of lesions is documented in both IgG control and anti-activin A antibody treated mice. (**D**) *Left panel:* Representative images (×10 and ×40 magnification) of liver tissue from control treated versus anti-activin A treated KPC mice stained with H&E (upper panel) or ductal marker protein CK19 (Lower panel). *Right panel:* Quantification of distant metastatic formation in the liver tissue of control (n = 5) and treated (n = 7) mice. Statistical analysis unpaired Student t-test (ns: not significant).

To evaluate the role of activin A in metastasis in vivo we utilized both KPC and KPC^luc^ mice generated as previously described^35,36^ and outlined in Figure S3A. The formation of tumors in the KPC^luc^ mice was followed by live imaging of the luciferase bioluminescence in cre positive cells targeted to the pancreas by the Pdx-1 promoter. This allowed visualization of cells that underwent cre recombination (pancreatic tissue and tumors) over the time course of the anti-activin A antibody treatment (Figure S3B). Pancreatic masses staining positively were visible in all mice. These mice were used in combination with KPC mice to examine inhibition of activin A in vivo using a neutralizing antibody. KPC mice (8–12 weeks of age) received weekly injections of either activin A-neutralizing antibody (n = 7) or IgG isotype control (n = 5) (Fig. 4A and Figure S4A). The half-life of serum IgG isotype monoclonal antibodies in adult mice was previously reported to be 6 to 8 days^37^. This time period was used successfully in our previous publications on activin A in pancreatitis^14,34^. Activin A levels in plasma collected at necropsy were determined by ELISA. Circulating activin A levels were lower in the mice treated with neutralizing antibody (0.3355 ng/ml ± 0.1381, n = 4) than in control-treated mice (2.113 ng/ml ± 0.5894, n = 4) (Figure S4B). Tissue sections from pancreas were stained for α-SMA, a stromal marker of activated HPSCs, and adjacent sections were stained for activin A. We observed co-localization of activin A staining in stromal cells of pancreatic tissue sections. The activin A-neutralizing antibody-treated mice displayed decreased activin A staining, indicating that the antibody was able to penetrate the dense stroma and reduce the activin A expression in the stromal cells of the tumor microenvironment (Fig. 4B). Pancreas tissue sections from these animals were assessed by H&E staining to quantify cystic neoplasms (CN) as defined by a cyst-like structure comprising a layer of epithelial cells surrounding a lumen. Intraductal papillary mucinous neoplasm (IPMN) cystic lesions, which occur with less frequency, were also quantified and identified by a glandular structure that typically has layers of cells, mostly well-differentiated, which can be filled with mucin and protrude into the narrow lumen. Both these types of pre-cancerous lesions are at risk for undergoing malignant transformation^38^. Mice treated with the neutralizing antibody expressed similar numbers of CNs and IPMNs as control-treated mice (Fig. 4C). The liver has been shown to be a preferential metastatic organ of PDAC disease^39^, therefore, we quantified the number of liver metastases in both control and neutralizing antibody-treated KPC mice. As assessed by histopathology by two independent pathologists, the IgG control KPC mice have a higher incidence of metastasis formation. This was confirmed by CK19 staining in the liver (Fig. 4D), indicating the presence of pancreatic ductal cells in the livers of control mice but not in the majority of anti-activin A-treated mice. These data indicate that the inhibition of activin A by the neutralizing antibody did not impede formation of the pre-cancerous lesions but was able to inhibit metastasis in four of five treated mice.

In summary, our data support that activin A is produced in the stromal cells of the pancreatic tumor microenvironment. The dense stromal tumor microenvironment contributes to increased activin A, which acts on the tumor epithelial cells to promote EMT, cell migration and metastasis. Therapeutic inhibition of activin A may be efficacious in a sub-group of PDAC patients with high stromal activin A staining.

## Discussion

Earlier studies focused on the mutations in PDAC tumor cells for potential therapeutic targets^40^, however, this line of research has not yielded new therapeutic approaches. Recognizing this lack of progress, current studies to identify therapeutic targets are focusing on the interaction of tumor cells with stroma and the role of stroma in PDAC progression^41^. In this study, we observed that human stromal-enriched PDAC cases express higher levels of activin A protein, which correlates with worse prognosis and reinforces the need to study tumor-stromal interactions. In cultured pancreatic cell types, we observed elevated levels of activin A in the human pancreatic stellate cells (HPSC). As disease progresses, the tissue architecture becomes less acinar-like and more stroma-dense, which could be an underlying source of elevated activin A levels in tissue and serum of patients with advanced PDAC. A subset of high tumor-burden PDAC mice (KPC) had increased activin A levels in plasma. We observed that in vitro activin A did not increase tumor cell growth, but rather increased cell migration. Properties of local invasion, including ectopic to some organs, is in many ways a much simpler process than metastasis, with the main similarity being the breach of the basement membrane. Beyond that, metastasis requires EMT, intravasation, immune system evasion, extravasation and survival in a foreign environment. Furthermore, pancreatic cancer has the propensity to metastasize even when the primary tumor is small, particularly in humans. In mice, the presence of circulating tumor DNA and other blood markers can signal the presence of cancer even before a gross tumor is visible, as shown in KPC mice^42,43^. Because inhibition of activin A in vivo was shown not to reduce tumor burden in our mouse cohort, it instead indicates that it reduced and/or delayed metastasis formation via reduction of activin A in the pancreatic stromal cells and in the plasma of KPC mice.

The tumor microenvironment of PDAC is highly dynamic and displays remarkable heterogeneity and complexity of its multiple components^6^. The pancreatic stroma has been regarded as protective in some cases by reducing vascularity into the solid tumor^44^, and, at the same time, it has been described as promoting cancer-cell heterogeneity and shaping the tumor architecture to aid tumor progression^45^. This heterogeneity is mirrored in our data, which identify a sub-group of stromal-rich PDAC. This subgroup expresses increased activin A and has a worse prognosis in comparison to epithelial-rich PDAC. Various genomic approaches have been applied to identify sub-groups of PDAC patients to employ precision medicine approaches and more efficacious treatments based on disease biology^46^, however, the contribution of stroma has only been appreciated recently. Nicolle et al. utilized both surgical tissue as well as biopsy from unresectable tumors to develop patient derived xenografts (PDX) in immune-compromised mice^8^ and were able to distinguish between tumor expression (human genes) versus stromal expression (mouse genes). Similar to the in silico study of Moffitt et al.^47^, Nicolle et al. identified two tumor profiles, basal and classical, as well as two stroma subtypes, activated and normal; the basal tumor and activated stroma subtypes correlated with worse prognoses^8^. To validate their model system, Nicolle et al. analyzed gene expression in pathways known to be involved in tumor/stroma cross-talk and measured greater expression of INHBA (activin A) in activated stromal versus tumor tissue and greater expression of ACVR2B (a component of the activin receptor) in tumor tissue than in stroma^8^. Our immunohistochemical identification of a stromal-dense activin A protein-expressing sub-set of tumors in the human PDAC TMA is in line with Nicolle’s pancreatic cancer PDX gene expression results.

Pancreatic cancer is commonly diagnosed when disease is already advanced, resulting in poor outcomes^48^ due to a lack of effective treatments. Therefore, understanding metastasis may identify druggable pathways to enhance the efficacy of current treatments^49^. We have previously reported that stromal cell-secreted activin A^14^ and increased stiffness increase cell migration in colon cancer cell lines^13^. Here we report that activin A increases wound closure and cell migration of PDAC cell lines. We previously reported that activin A was growth-suppressive in colon cancer cells^21,22^ and here we did not observe an activin A-induced increase in cell number. This is in contrast to the growth promoting effects on PDAC cells as reported by Togashi^26^, perhaps because we used PDAC cell lines expressing low levels of activin A. Importantly, systemic treatment of KPC mice expressing pancreatic tumors resulted in a decrease in pancreatic expression of activin A and a loss of metastases to the liver in four of five treated mice.

As a caveat to our in vivo study, the data from our KPC cohort had a high variance. Ideally, we initiated our treatment protocol after the formation of tumors, but before tumor metastasis, however, a weakness in the KPC model is the variation in the time frame for primary tumor formation; therefore, we had to generalize a time frame in which to initiate treatment. We attempted to address this issue through the introduction of the luciferase gene in cre positive cells to image tumor formation^35^, however, even with this additional marker, visualization of tumors by bioluminescent screening was not straightforward. Indeed, an optimal therapeutic window may have been prohibitive in some animals due to this variation and may have masked contribution to an observed survival benefit through the inhibition of activin A. In addition, large primary tumor size is likely a predominant factor in animal death due to impingement on critical ducts and organs. Therefore, the addition of a growth-inhibiting therapeutic, such as gemcitabine^50^, along with inhibition of activin A may be more efficacious. Our data indicate that inhibition of activin A does not decrease tumor growth but does decrease metastasis. This leads us to postulate that a therapeutic approach that inhibits activin A may be more successful in humans where the main cause of death is due to metastases.

Our data indicate that inhibition of activin A could benefit patient survival by decreasing the most common cause of death in PDAC, namely, metastasis. One such inhibitor for activin signals is sotatercept (ACE-011), an activin receptor inhibitor (ActR11A-IgG1Fc) that is in clinical trial Phase II for the treatment of chemotherapy-induced anemia in patients with metastatic non-small cell lung cancer^51^. This trial has demonstrated that the administered antibody is active and has an acceptable safety profile in humans^52^. The route of administration of ACE-011 may be clinically favorable as administration of the antibody is via injection every 28 days (NCT01562405), versus the more frequent administration of chemotherapeutics^53^.

Taken together, these data suggest that stromal activin A secretion is a contributor to PDAC disease progression and is a predictor of worse prognosis and disease progression. In advanced PDAC, there were less normal-like structures and acinar cells in pancreatic tissue and an increased presence of stroma and fibrosis when activin A secretion was high. Our KPC mouse data indicate that activin A expression in the blood reflects the protein expression observed in PDAC stroma. Indeed, serum activin A levels may serve as a biomarker to identify the stromal presence of activin A and serve to risk-stratify patients at the clinic with a more mesenchymal PDAC subtype. Activin A signaling shows promise as a treatment target for a subset of PDAC patients who have already developed a tumor and show high activin A levels in serum. Inhibiting activin A could reduce metastatic cell potential and provide a better survival profile than current chemotherapeutics.

## Methods and materials

### Chemicals and reagents

Human recombinant activin A protein (R&D Systems; MN, USA) was reconstituted according to manufacturer’s instructions. Anti-activin A neutralizing antibody mAb3381 (R&D Systems; MN, USA) was resuspended in PBS at 0.5 mg/ml, aliquoted and stored at − 20 °C. Recombinant Follistatin 288 protein (R&D Systems, MN, USA), was used at 100 ng/ml in cell culture in vitro and was added 30 min prior to activin A. Follistatin was used in preliminary experiments as a positive control for activin A inhibition to confirm the inhibitory actions of the activin A neutralizing antibody.

### Antibodies

Antibodies include: Anti-INHBA (IHC) from Ansh Labs (TX, USA), anti-activin A (neutralizing, mAb3381) from R&D Systems (MN, USA); anti-α-SMA (ab19245), anti-vimentin (3390S), anti-pSMAD2 (3104S) and anti-pSMAD2/3 (S465/S467; 8828S) from Cell Signaling (MA, USA); anti-CK19 from University of Iowa Hybridoma Bank (IA, USA). Donkey anti-rabbit, sheep anti-mouse or donkey anti-goat antibodies conjugated to horseradish peroxidase (Amersham Biosciences; Little Chalfont, UK), DAKO anti-rabbit and anti-mouse, and Vector anti-rat were used as secondary antibodies for IHC staining. Secondary antibodies anti-mouse and anti-rabbit Alexa Fluor 594 and Alexa Fluor 488, and donkey anti-goat-Alexa Fluor 594 were purchased from Invitrogen.

### Human samples

PDAC cancer tissue microarray (TMA) slides with outcomes data were obtained from US Biomax, Inc (Rockville, MD) with 63 PDAC samples of cancer and adjacent-normal tissue stained with antibody against the activin A subunit INHBA (Ansh lab LCC, Webster, TX). Slides were blindly scored by two independent investigators using a four-point scoring system from zero for no staining to three for strong staining. All clinical variables provided by US Biomax, Inc, (age, gender, stage, overall survival) were independently analyzed and correlated to activin A protein-staining level. TMA slides were Hematoxylin & Eosin (H&E) stained to determine stromal versus epithelial components and the average between the investigators was computed.

### Murine samples

Age-matched animals were sacrificed and subjected to pathological examination of the pancreas, colon, duodenum, liver, spleen, lungs, mesentery and skeletal muscle. Tissues sections were either stained with H&E immunohistochemistry (IHC) or immunofluorescence (IF) using previously described protocols^22,54^.

### In silico analysis

Oncomine dataset^28^ was queried to evaluate activin A mRNA transcript expression in three human PDAC or pancreatic cancer (PC) studies in cancer tissue and its matching adjacent-normal tissue. Oncomine data from Badea, Pei and Logsdon were analyzed and measured in terms of fold change (log^2^ values) and averaged tumor tissue values were compared against corresponding adjacent-normal tissue for each independent study.

### Cell culture

Pancreatic cancer cell lines PANC-1 and MIA PaCa-2 were purchased from American Type Cell Culture (ATCC, Manassas, VA). Human pancreatic stellate cell line (HPSC) was provided by Dr. Hwang. Cells were cultured as previously described in Dulbecco's Modified Eagle's Medium (DMEM) supplemented with 10% FBS^54^. All cells were serum-starved for 24 h prior to treatment. Activin A concentration for treatment was 25 ng/ml, as previously determined^14^.

### ELISA

Activin A Quantikine ELISA (R&D Systems) was used following the manufacturer’s instructions as previously described^14^. Cell culture supernatants were analyzed without freezing; murine plasma samples were analyzed after storage at − 80 °C.

### Cell viability assay

Cells were plated at a density of 5 × 10^4^ cells/mL and serum-starved for 24 h prior to treatment. Metabolic activity, as a surrogate of cell growth, was determined by the wst-8-based Cell Counting Kit-8 (Dojindo Molecular Technologies, Inc., Rockville, MD) as previously described^14^.

### Wound healing and transwell migration assay

4 × 10^5^ cells were grown to confluence, and serum-starved for 24 h. Confluent monolayers were disrupted by a scratch with a 10 μL pipette tip. Media supplements were added as indicated and images were captured with a brightfield microscope at increasing time points with 10× magnification field. Wound area was calculated with ImageJ and compared to initial scratch area and percentage wound closure was calculated. For the transwell migration assay, 3 × 10^4^ cells were then placed in a 12 mm transwell as previously described^21^. Following 24 h of serum-starvation, the treatments were added as noted in the figure legend (2 mg/ml anti-activin A antibody, PBS or 25 ng/ml activin A). After 12 h the migrated cells were fixed and mounted on a slide with 4′6-diamidino-2-phenylindole (DAPI) (Vector Laboratories). Pictures of five different fields per slide were taken using DAPI channel in the fluorescent and quantified by ImageJ software using Wound Healing macro.

### Immunocytochemistry

Cells were cultured on 8-well chamber slides, treated as described in the figure legends and processed as previously described^21^. Images were obtained with microscope NIKON Eclipse Ti with a NIKON DS-Qi2 camera and NIS-Elements AR software (Tokio, Japan). Filters were DAPI, FITC and Texas red. Exposure for 5 s for green channel (vimentin 488), 7 s for Red channel (activin A 594) and 4 s for DAPI (blue channel). Signal threshold: 3000 for Green channel (vimentin 488), 4500 for Red channel (activin A 594) and 14,000 for DAPI (blue channel). Gamma 0. Gain 1. Resolution: 1600 × 1200. Downstream process: − 20 Contrast on Powerpoint on every picture.

### Immunoblotting and antibodies

Immunoblotting was performed as previously described^55^ with the following antibodies: mouse monoclonal to pSMAD2 3104S (Cell Signaling, MA, USA) and mouse monoclonal GAPDH sc-47724 (Santa Cruz, TX, USA). Protein bands were visualized by chemiluminescence detection by BIO-RAD ChemiDoc MP Imaging System (Thermo Scientific, MA, USA).

### In vivo murine pancreatic cancer model

Generation and characterization of Pdx-1-Cre;LSL-Kras^G12D/+^;LSL-p53^R172H/+^ (KPC) mice in a C57BL6 background, a subset of those mice that expressed the luciferase allele in cre positive cells (Pdx-1-Cre;LSL-Kras^G12D/+^;LSL-p53^R172H/+^;LSL-ROSA2^Luc/+^ ;KPC^luc^) have been previously described^35,36^. The transgenic schema is illustrated in Figure S3A. Both sexes of mice were used at 8–12 weeks after birth; mice bearing tumors were treated weekly with either IgG control or anti-activin A neutralizing antibody at a concentration of 2 mg/kg via intraperitoneal injection. The once weekly injection of the IgG antibodies was based on the previously reported observation that the half-life of serum IgG isotype monoclonal antibodies in adult mice is 6 to 8 days^37^ KPC^luc^ mice were kindly provided by Dr. Sam Grimaldo at University of Illinois Chicago. Mice were euthanized after 13 weeks of treatment or when mice became moribund. Mice were provided cob bedding and paper for nesting and had ad libitum access to chow and water.

A standard animal tissue collection included pancreas, liver, duodenum, spleen, colon, skeletal muscle, lungs and in some cases lymph nodes and mesentery. Tumor volume was measured by caliper and the formula V = (W^2^ × L)/2, where V is “volume”, W is “width” and L is “length”. Whole animal and dissected wet pancreas weights were recorded. Tissues were formalin-fixed and paraffin-embedded for histological analysis, preserved in RNA-later for RNA studies or snap-frozen for protein analysis and kept at − 80 °C. All mouse experiments were reviewed and approved by the University of Illinois at Chicago (UIC) Institutional Animal Care and Use Committee. Interventions were performed during the light cycle. All methods were performed in accordance with the UIC Institutional Animal Care and Use Committee guidelines and regulations. The study was performed in compliance with the ARRIVE guidelines.

### In vivo imaging

Anesthetized animals were injected intraperitoneally with 15 mg/kg d-luciferin and placed on IVIS imager (Caliper, PerkinElmer). Images were acquired starting 10 min after injection. Signal was detected using the IVIS Lumina II CCD camera system and analyzed with the Living Image 2.20 software package (Caliper Life Sciences). Intensity of bioluminescence was color-coded for imaging purposes (Figure S3B).

### Statistical analysis

The data were analyzed by GraphPad Software and either t-test, one-way ANOVA or two-way ANOVA as indicated. Results were arranged by the Tukey method and were considered significant at p < 0.05. In vitro results are presented as ± SD and in vivo results are presented as mean ± SEM unless otherwise noted. A minimum of n ≥ 3 for all experiments.

### Study approval

All experiments involving the use of mice were performed following protocols approved by the Institutional Animal Care and Use Committee at the UIC and in compliance with the ARRIVE guidelines. PDAC tissue microarray was commercially purchased from US Biomax Inc.

## Supplementary Information

Supplementary Information.

## Data Availability

All data generated or analyzed during this study are included in this published article (and its Supplementary Information files).
