# Supplementary material for: Role of stromal activin A in human pancreatic cancer and metastasis in mice
Source: Sci Rep. 2021 Apr 12;11:7986. doi: 10.1038/s41598-021-87213-y (PMC8042028; doi:10.1038/s41598-021-87213-y)
Supplement: Supplementary file 1 — Supplementary Information. [file 41598_2021_87213_MOESM1_ESM.pdf]

Role of stromal activin A in human pancreatic cancer and metastasis in mice

Georgina Mancinelli[1], Carolina Torres[1], Nancy Krett[1], Jessica Bauer[2], Karla Castellanos[1], Ron McKinney[1], David Dawson[3], Grace Guzman[4], Rosa Hwang[5], Sam Grimaldo[6], Paul Grippo[1]*, Barbara Jung[2]*

Grant Support: NIH grant R01 CA 141057 to BJ

Corresponding author:

Barbara Jung, M.D. (bhjung@uw.edu)

Professor and Chair, Department of Medicine

1959 NE Pacific Street, RR-512

Box 356020

Seattle, WA 98195-6420

Phone: 206-543-3293

FAX: 206-543-3947

Author affiliations:

[1]Department of Medicine, Division of Gastroenterology and Hepatology, University of Illinois at Chicago, Chicago, IL, USA

[2]Department of Medicine, University of Washington College of Medicine, Seattle, WA, USA

[3]Ronald Reagan UCLA Medical Center, UCLA Medical Center, Santa Monica, CA, USA

[4]Department of Pathology, University of Illinois at Chicago, Chicago, IL, USA

[5]Department of Breast Surgical Oncology, Division of Surgery, The University of Texas MD Anderson Cancer Center, Houston, TX, USA

[6]Department of Surgery, University of Illinois at Chicago, Chicago, IL, USA

* These authors contributed equally to this work.

Supplemental Figures
Figure S1.

A

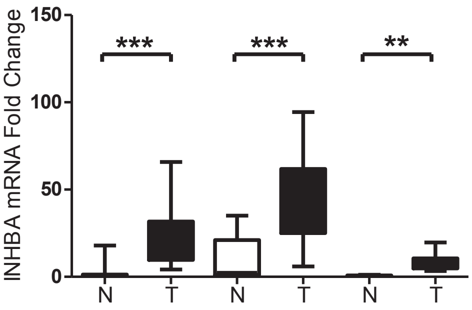

B

Pathology diagnosis	Number
Adenosquamous carcenoma	3
Ductal carcinoma	60
NAT (normal adjacent tissue)	57
**Gender**	
Female	27
Male	36
**Grade**	
I	1
I-II	1
II	25
II-III	19
III	7
**Stage**	
1A	1
1B	4
2A	23
2B	22
4	4
**Survival status**	
Alive	normal 13
Deceased	50
**Survival months**	
0-10	34
11-20	11
21-30	7
31-40	7
41-50	1
51-60	2
61-70	1

C

Scoring	Staining intensity
0	no staining
1	weak
2	medium
3	high

D

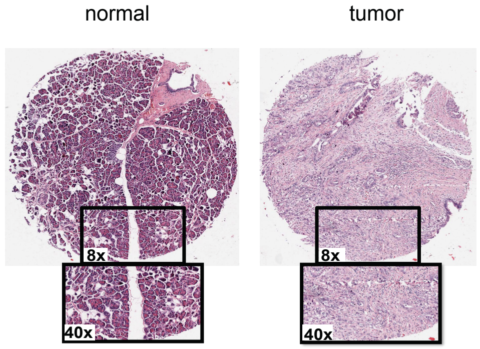

Figure S1. Activin A is expressed in human pancreatic tumors. A) In silico analysis for Oncomine data base. INHBA mRNA expression from adjacent normal (white bars) compared to pancreatic tumor tissue (black bars) from studies reported by Badea et al (n=39 N, n=39 T); Pei et al (n=16 N, n=36 T) and Logsdon et al (n=5 N, n=10 T). Data are expressed as mean ± SEM, Student t-test. B) Clinical data from human pancreatic tissue TMA. C) Activin A immunohistochemistry staining intensity score for TMA.
D) TMA representative H&E staining of normal adjacent and tumor cores from the same patient. Normal tissue is comprised of acini, ductal and islet cells with minimal connective tissue. Corresponding tumor section show loss of tissue architecture, immune cell infiltration, predominant reactive stroma, loss of acini and ductal cell morphology, loss of basal cell polarity and an increase in cells undergoing mitosis.

Figure S2.

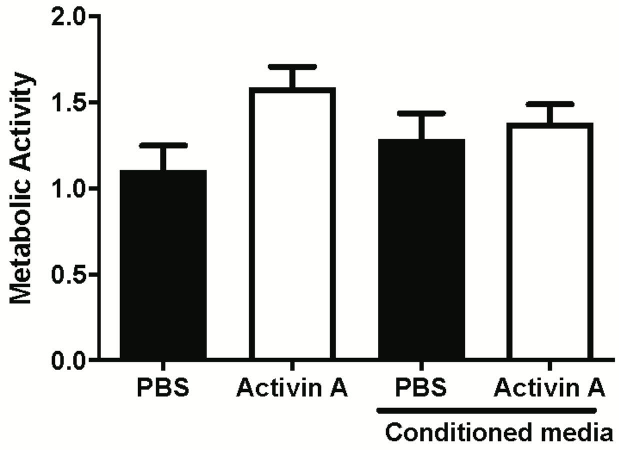

Figure S2. Validation of anti-activin antibody neutralization of activin signaling in vitro. Neither exogenous activin A nor stromal secreted activin A stimulate metabolic activin in the MIA PaCa-2 epithelial pancreatic cell line. Control treatment (PBS, Black bars) and activin A treatment (25 ng/ml, white bars).

Figure S3.

A

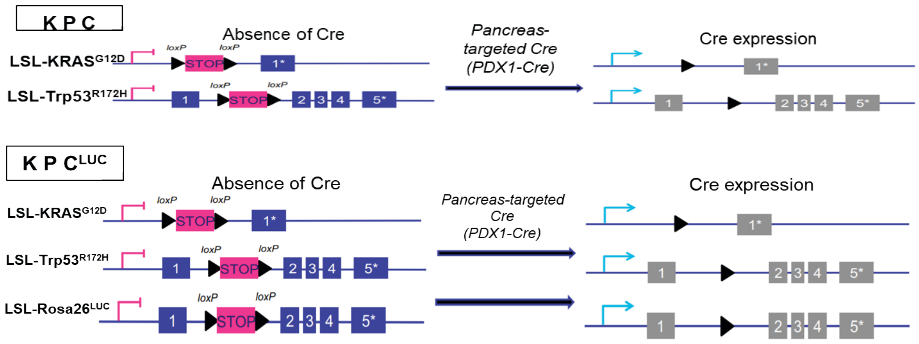

B

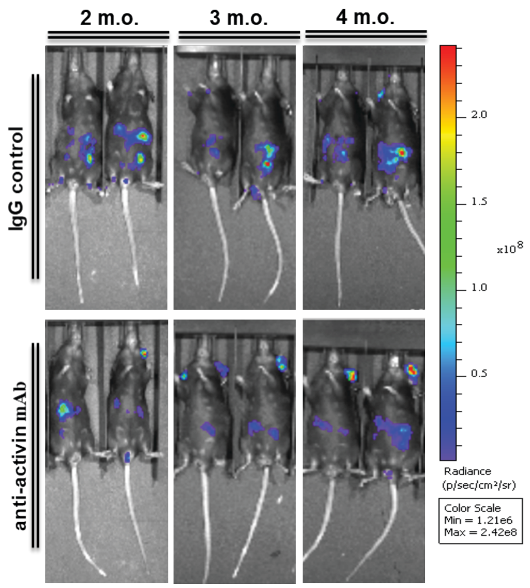

Figure S3. In vivo KPC pancreatic cancer mouse models. A) KPC mice harboring mutations in the KRAS and Tp53 endogenous alleles upon cre recombination in cells expressing the PDX-1 transcription factor were bred in our mouse colony. KPCluc mice harbor the same mutations in addition to expression of luciferase in PDX-1 expressing cells upon cre recombination allowing visualization of pancreatic tissue mass. B) Pancreatic cells expressing luciferase in vivo in KPCluc mice. Mice at 2 months, 3 months and 4 months of age were injected with luciferin (15mg/ml) i.p. and anesthetized with isoflurane before bioluminescence live imaging. Images were captured 10 minutes after injection with a delay of 30 between images. Treatment with the activin A neutralizing antibody was initiated at 2 months of age.

Figure S4.

A

Control mice	Treated mice
n=5	n=7
~ 8-12weeks of age	~ 8-12weeks of age
2mg/kg IgG	2mg/kg anti-activin mAb (MAB3381, R&D Systems)

B

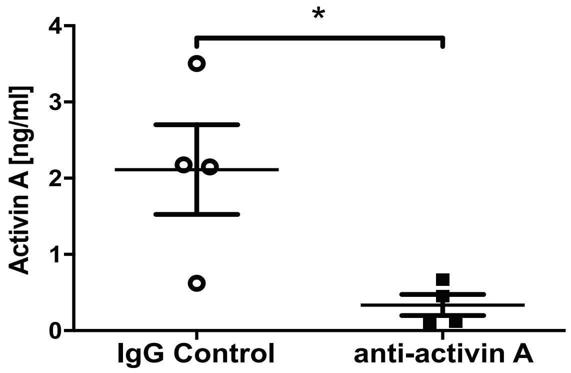

Figure S4. Pancreatic plasma and tissue shows reduction of activin A with anti-activin A mAb treatment. A) Schema of treatment protocol of KPC mice. B) Activin A levels in KPC mouse plasma is reduced by treatment of anti-activin A mAb in comparison to animals treated with the IgG control Ab. Mean ± SEM unpaired Student t-test using Graph pad (*p<0.05).
